# Structure and Conformation of the Carotenoids in Human Retinal Macular Pigment

**DOI:** 10.1371/journal.pone.0135779

**Published:** 2015-08-27

**Authors:** Ana-Andreea Arteni, Mathias Fradot, Denise Galzerano, Maria M. Mendes-Pinto, José-Alain Sahel, Serge Picaud, Bruno Robert, Andrew A. Pascal

**Affiliations:** 1 Institute for Integrative Biology of the Cell (I2BC) & Institut de Biologie et de Technologies de Saclay, CEA, UMR 8221 CNRS, Université Paris Saclay, Gif-sur-Yvette, France; 2 INSERM, UMR S968, Institut de la Vision, Paris, France; University of Western Australia, AUSTRALIA

## Abstract

Human retinal macular pigment (MP) is formed by the carotenoids lutein and zeaxanthin (including the isomer meso-zeaxanthin). MP has several functions in improving visual performance and protecting against the damaging effects of light, and MP levels are used as a proxy for macular health–specifically, to predict the likelihood of developing age-related macular degeneration. While the roles of these carotenoids in retinal health have been the object of intense study in recent years, precise mechanistic details of their protective action remain elusive. We have measured the Raman signals originating from MP carotenoids in *ex vivo* human retinal tissue, in order to assess their structure and conformation. We show that it is possible to distinguish between lutein and zeaxanthin, by their excitation profile (related to their absorption spectra) and the position of their ν_1_ Raman mode. In addition, analysis of the ν_4_ Raman band indicates that these carotenoids are present in a specific, constrained conformation *in situ*, consistent with their binding to specific proteins as postulated in the literature. We discuss how these conclusions relate to the function of these pigments in macular protection. We also address the possibilities for a more accurate, consistent measurement of MP levels by Raman spectroscopy.

## Introduction

The *macula lutea* is an oval-shaped, highly-pigmented region (diameter 5–6 mm) near the centre of the retina of humans and other primates, the “yellow spot” first described in the 18^th^ Century [1]. It is responsible for sharp, clear central vision and the ability to perceive colour. The yellow pigmentation of the macula is due to the presence of the dietary carotenoids lutein and zeaxanthin (including the isomer meso-zeaxanthin). Their functions are multiple–reduction of “haze” effects; reduction of low-light glare; and protection against age-related macular degeneration (AMD, the principal cause of blindness in old age).

The carotenoids of the macular pigment (MP) in human retina are mainly concentrated in the central foveal region, an area about 1.5 mm in diameter with a high density of cone photoreceptors, enabling high acuity colour vision. Indeed, in spite of significant variation from one subject to another, in most cases the MP concentration increases steadily in towards the centre of the fovea [2]. When viewed in cross section, MP is located anterior to the photoreceptor outer segments and the retinal pigment epithelium [3,4]. This location underlies a number of their functions. The two “visual” roles are directly related to their absorption properties (*i*.*e*. their colour)–they serve as filters of blue light, which is predominantly responsible for hazing and glare artefacts (as light scattering is inversely proportional to the fourth power of wavelength). This filtering effect is also thought to provide a degree of protection against light-induced damage, by shielding these vulnerable tissues from short-wavelength photons (blue and UV light; shorter wavelength = higher energy). MP is also considered for a systemic anti-inflammatory function, of particular relevance as AMD displays features of a chronic low-grade systemic inflammatory response [5].

While the filtering effect of the macular carotenoids may significantly reduce light-induced photodamage of the underlying retinal tissue, this protection cannot be total as some high-energy photons will always get through (and indeed, not all of the blue light should be filtered out as otherwise this would itself impair colour vision). It therefore seems likely that the focussing of light at the macula, and particularly the central fovea, will inevitably lead to some unwanted photochemical reactions occurring. The specific uptake of lutein and zeaxanthin by the macula, to the exclusion of other dietary carotenoids [6], should probably be interpreted with this in mind. Indeed, the specific role of both carotenoids in a number of (photo)protective mechanisms in nature has already been revealed. As for most carotenoid molecules, they are efficient scavengers of reactive oxygen species [7], but, more specifically, both zeaxanthin and lutein are at the basis of the major mechanisms protecting plants against light stress–quenching of chlorophyll singlets [8,9] and triplets [10], and preventing lipid peroxidation [11].

The protective roles of carotenoids in photosynthetic tissues are enhanced by the binding of these pigments to protein hosts [12]. This is thought to be due to a number of factors–the need to hold the carotenoid at a specific distance and orientation relative to the quenched “substrate” (*e*.*g*. triplet chlorophyll); the prevention of carotenoid degradation upon its elevation to a higher energy level (often occurring through isomer formation); and the possibilities that protein-binding present for tuning of the protective function. It is therefore of interest, when considering the possible protective roles of the macular pigment, that a number of recent studies have indicated that these carotenoids are also bound by specific host proteins within the retinal tissue [13], and evidence has been presented that the binding of zeaxanthin does indeed enhance its anti-oxidant function [14]. It is thought that the same macular proteins mediate the retinal capture of these carotenoids from serum lipoproteins [15,16].

**Fig 1:**
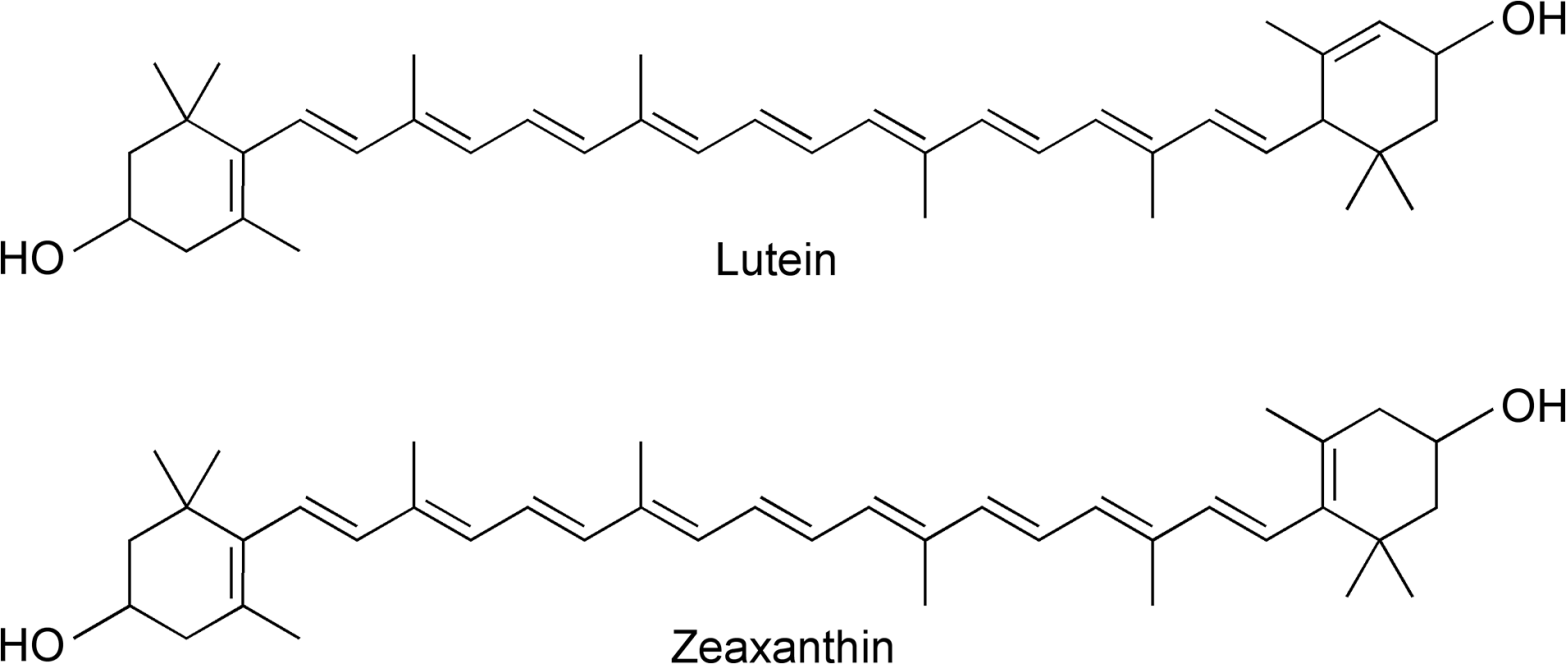
Chemical structures of lutein and zeaxanthin.

Of central importance to the function(s) of carotenoids is the electronic structure of their conjugated C = C chain, which is at the basis of their photoactivity. In recent years, resonance Raman has emerged as an invaluable tool to study the structural and functional properties of carotenoid molecules [17,18]. Resonance Raman spectroscopy has previously been used as an objective optical approach for the detection of carotenoids in living human tissue, including the skin and the retina [19,20]. These previous studies mainly involved only the detection and quantification of the carotenoid present. Resonance Raman spectroscopy can potentially provide a much greater level of detail, both in discriminating the different carotenoid species in a mixed population, and by providing a detailed analysis of their molecular configuration and conformation. In resonance Raman, the Raman signal is enhanced dramatically (by up to six orders of magnitude) when the excitation wavelength used matches the position of an electronic transition (absorption band) of the scattering molecule. This allows for measurements in complex media–*e*.*g*. on pigmented cofactors such as carotenoids bound to their protein hosts, and even *in vivo*. Moreover, the intensity of the resonance Raman signal depends directly on the relative positions of the excitation wavelength and the electronic transition of the scattering molecules. Thus for a sample containing several carotenoid populations, each possessing slightly different absorption properties, the resonance intensity of each of the species will be slightly different at each excitation wavelength used–any given species will dominate the spectra for excitations closer to its own absorption band. Analyzing an ensemble of spectra obtained at different wavelengths may thus yield information on each of the molecular species constituting the mixed population. Note that this is true not only for chemically-different carotenoid molecules, but also for chemically-identical carotenoids with different absorption properties (due to differential tuning by their environment). For instance, the two lutein molecules bound to the major higher plant light-harvesting protein can be observed selectively because their absorption bands are shifted relative to one another [18]. This should also be the case here for lutein and zeaxanthin, as their different conjugation lengths (see Fig 1) result in different absorption properties *in vitro* [21] and in the *macula* [22,23].

**Fig 2:**
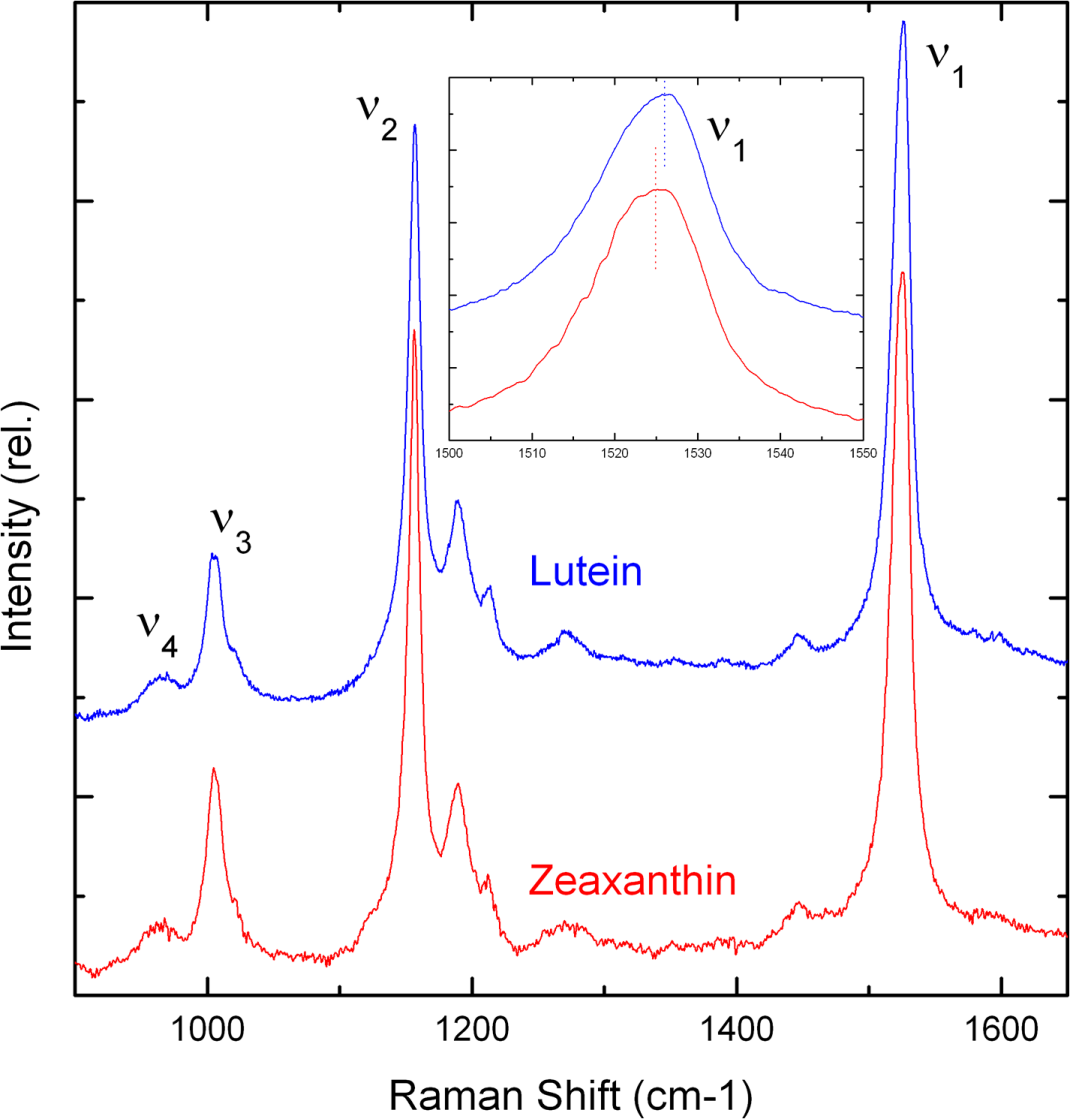
Resonance Raman spectra of MP carotenoids *in vitro*. Room temperature spectra in the 900–1650 cm^-1^ region are shown for zeaxanthin (red) and lutein (blue) in THF, excited at 488.0 nm. Inset: detail of the ν_1_ region.

Resonance Raman is a vibrational spectroscopy, giving precise molecular details of structure, interactions and environment for the molecule under study. The resonance Raman spectra of carotenoid molecules usually comprise four groups of bands, termed ν_1_ to ν_4_ (see Fig 2). The ν_1_ band, arising from conjugated C = C stretching modes, yields direct access to the extent of the carotenoid conjugated chain. Together with the structure of ν_2_ (*i*.*e*. how many satellites are observed in this spectral region), it may also be used to determine the molecular configuration (*trans*/*cis*) of the scattering carotenoid. Additional information can be obtained through study of the ν_4_ band, around 950 cm^-1^, which arises from out-of-plane motions of the H nuclei along the conjugated chain. In perfectly planar molecules, these modes are not coupled with the electronic transition (which is oriented along the plane of the molecule) and as a result they exhibit no resonance enhancement—the intensity of this band is thus extremely weak. However, ν_4_ gains intensity when the carotenoid is distorted out of the plane, *e*.*g*. due to steric hindrance within a protein binding pocket. The intensity of this band thus yields information about the planarity of the carotenoid molecule.

In this paper, we show that it is possible to discriminate between the different carotenoids constituting MP using resonance Raman excited at different wavelengths. Furthermore, the spectra provide details of the conformation of the MP carotenoids *in situ* (in *ex vivo* retinal tissue). These results are discussed in terms of the possible roles of the macular carotenoids in retinal protection, as well as the possibilities for using Raman spectroscopy for *in vivo* analysis of the macular pigment.

## Materials and Methods

### Ethics statement

Human eyes were collected and processed by the Euro Tissue Bank, Amsterdam (http://www.eurotissuebank.nl) in compliance with the European Directives for the processing of human tissues and cells. Donor globes were obtained 24 to 48 hours post-mortem, after corneas had been removed for transplantation, and all experiments were in accordance with approval DC-2008-346 from the French Ministry for Higher Education and Research.

### Sample preparation

Isolated lutein and zeaxanthin were obtained as described previously [24]. Retinal tissue from 8 subjects was harvested for the study after removing the iris, lens, ciliary body, anterior sclera and most of the vitreous. The macula was localized and trepanned with a 10 mm circular punch. Harvested macula and peripheral retina were separated from the underlying retinal pigment epithelium and overlying vitreous, and placed on glass microscope slides for use in Raman and confocal Raman measurements. Where necessary (for room temperature measurements), samples were de-oxygenated in an oxygen-free atmosphere and sealed under a cover-slip in order to avoid laser-induced photo-oxidation.

### Spectroscopic measurements

Resonance Raman spectra were measured using a Jobin Yvon U1000 Raman spectrophotometer equipped with a front-illuminated, deep-depleted charge-coupled device detector (Synapse Horiba, Jobin Yvon, Longjumeau, France). Where stated, samples were maintained at 77 K in a nitrogen-flow cryostat (Air Liquide, Sassenage, France). For macroscopic measurements on carotenoids *in vitro*, the signal was collected at 90° geometry. A confocal microscope with 10X objective was coupled to the Raman spectrometer for the analysis of retinal tissue (back-scattering geometry). Excitation wavelengths were provided by an argon laser (Coherent, Palo Alto, USA). Low intensity laser power was used to prevent degradation of the sample by the absorbed light energy (less than 20 mW for macroscopic measurements; around 20 μW through the microscope). Systematic comparison of the obtained spectra was performed throughout the time duration of each experiment to confirm sample integrity.

## Results

### Selective detection of carotenoid molecules in the macula

The structures of lutein and zeaxanthin are very similar, differing only in the length of their conjugated chain. While this chain extends into both cyclic end-groups for zeaxanthin, one of the ring double bonds is moved round one position in lutein so that it is not conjugated (Fig 1). It was recently shown that ring double bonds contribute about 0.3 effective C = C to the total chain length [17]. Accordingly, the expected conjugation length of lutein and zeaxanthin is 9.3 and 9.6, respectively. As the position of the (0,0) absorption transition of carotenoids is directly related to their conjugation length, this transition for zeaxanthin should be red-shifted relative to that of lutein by about 5 nm, for the pigments in the same conditions (same solvent, temperature, *etc*.) [17].

The resonance Raman spectra of lutein and zeaxanthin in tetrahydrofuran (THF) were obtained at room temperature for excitation at 488.0 nm (Fig 2). The spectra are almost identical, reflecting the similarity in their chemical structures discussed above. However, a clear difference appears in the frequency of their ν_1_ band (see inset to Fig 2). This band arises from C = C stretching modes, and directly correlates with the conjugation length of the scattering carotenoid molecule. ν_1_ is located at 1526.5 and 1525 cm^-1^ for lutein and zeaxanthin in THF, respectively. This frequency difference represents the principal distinguishing feature in spectra of the two carotenoids, which should also be observable in the intact retina. We therefore aimed to evaluate, for experiments conducted on retinal samples, whether or not we could distinguish these two carotenoids species in the intact macula.

**Fig 3:**
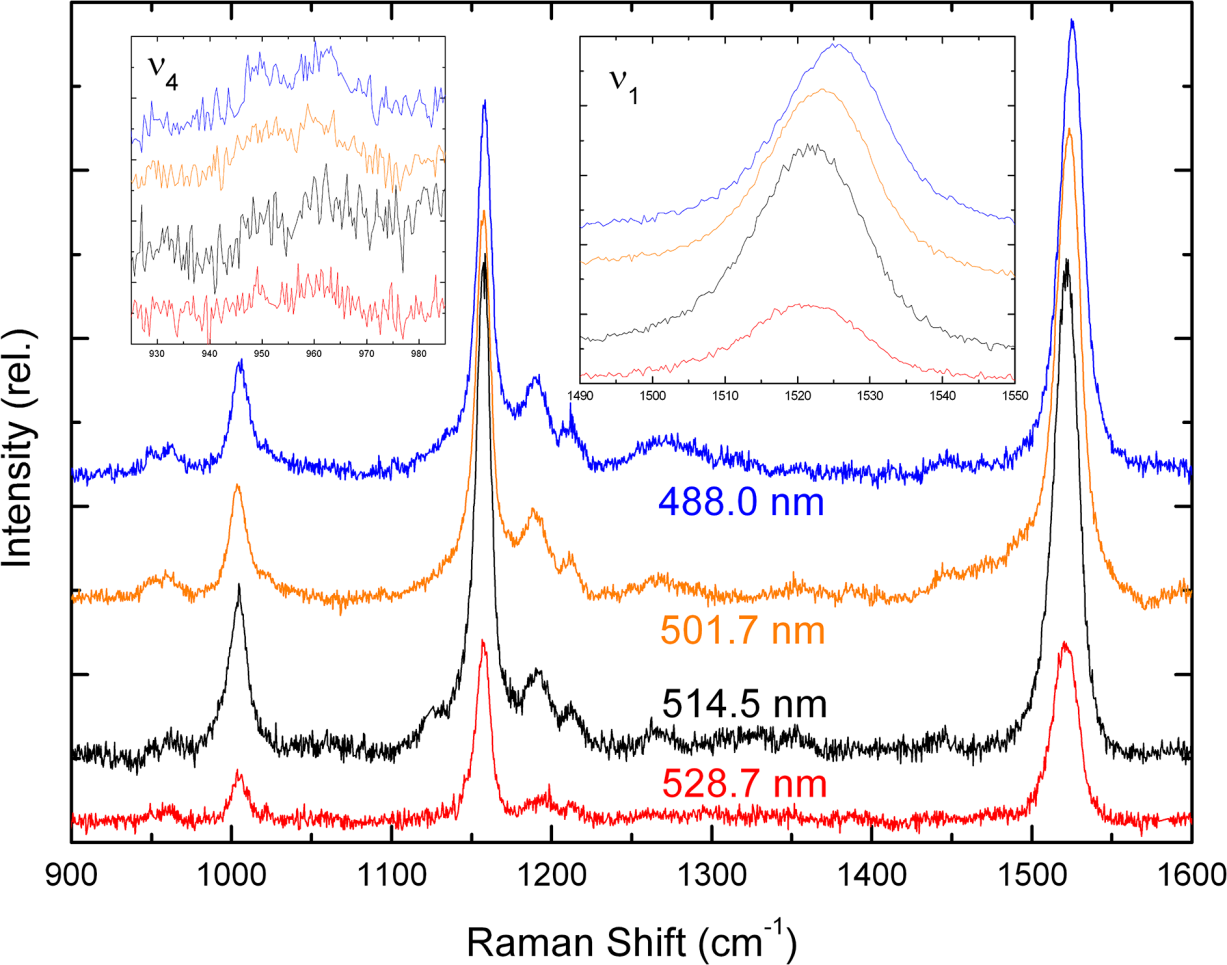
Resonance Raman spectra of human macula. Room temperature spectra (900–1600 cm^-1^) are shown for *ex vivo* human retina in the macular region, excited at 488.0, 501.7, 514.5 & 528.7 nm (blue, olive, black, red respectively). Details of the ν_1_ & ν_4_ regions are shown in the insets. Representative spectra are shown for a single macula, but were the same for all 8 subjects used in this study.

**Fig 4:**
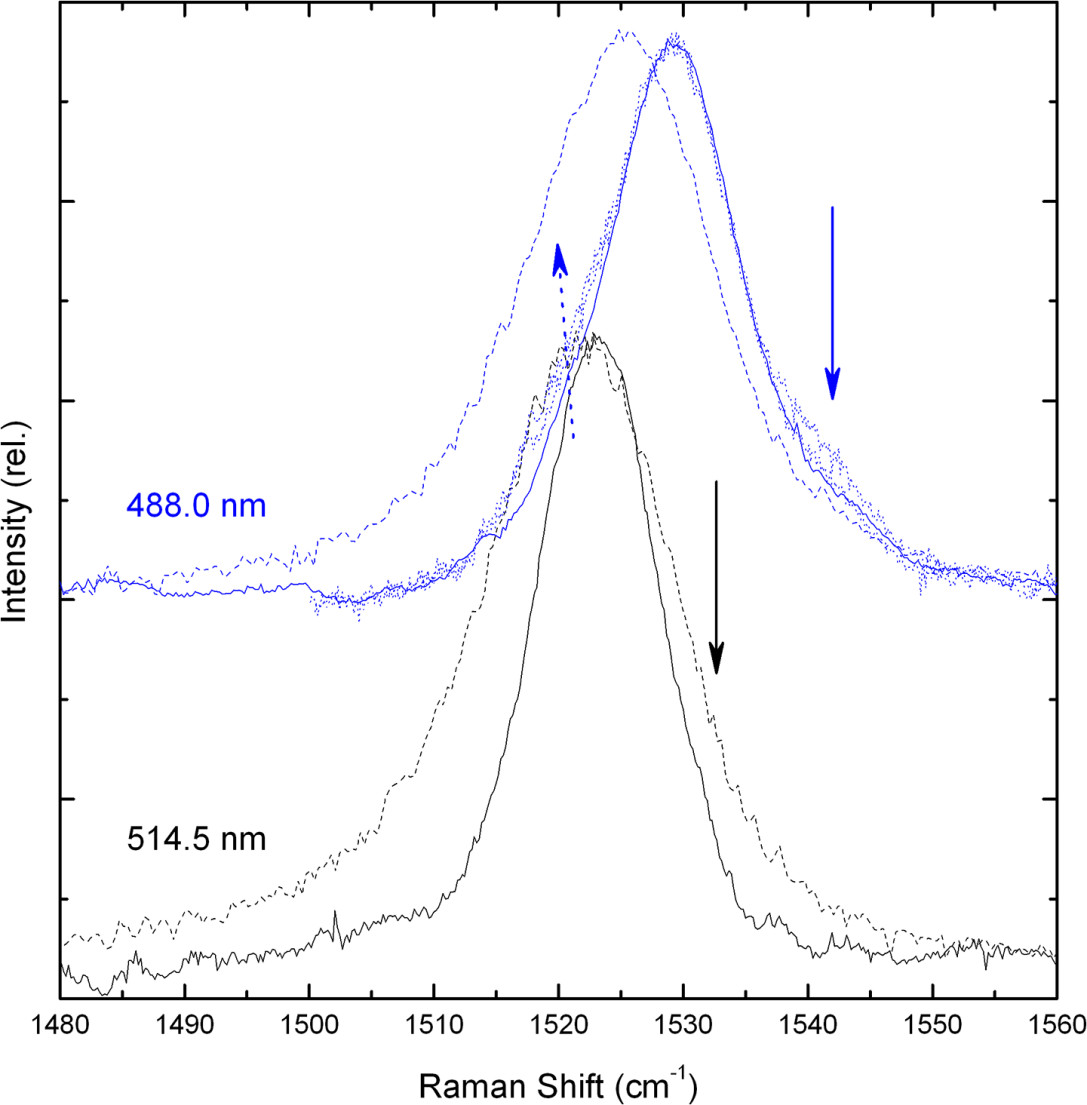
ν_1_ region of macula resonance Raman spectra. 77 K (solid lines) and room temperature (dashed lines) spectra are shown for excitation at 488.0 & 514.5 nm (blue, black respectively). For 488 nm, additional spectra are shown for measurements scanning in towards the foveal centre (dotted lines).

*Ex vivo* retina samples including the macular region were mounted for direct comparison of their resonance Raman spectra with those of lutein and zeaxanthin *in vitro*, and these spectra were measured at different excitation wavelengths (Fig 3). The spectra are broadly similar both to each other and to those of the isolated carotenoids. In the right-hand inset to Fig 3, a close-up of the ν_1_ region is shown. The ν_1_ band is clearly wider for the macular tissue than for the isolated carotenoids, particularly at 488 and 501.7 nm (half-width ~20 cm^-1^ compared to ~15 cm^-1^ for isolated pigments; see Fig 2). This indicates that both lutein and zeaxanthin contribute at all wavelengths, as expected from their small difference in absorption position. However, when shifting the excitation to higher wavelengths, the ν_1_ position shifts from 1525 cm^-1^ at 488 nm to 1521.5 cm^-1^ at 514.5 nm (Fig 3, right inset; see also Fig 4). This downshift in ν_1_ position indicates that at least two different carotenoid populations can indeed be distinguished in the macula, dominating alternately at different wavelengths. Given this shift in ν_1_ frequency and comparing with the spectra for the isolated carotenoids, we conclude that the lutein molecules present dominate at 488 nm, whereas zeaxanthin enters more into resonance as the excitation wavelength is increased. Indeed, given the increase in conjugation length for zeaxanthin, this carotenoid is expected to absorb slightly more to the red relative to lutein (see above) [17]. Note, in addition, that *in vitro* reconstitution of the hypothesised xanthophyll-binding protein for each macular carotenoid indicates a (0,0) absorption peak around 482 and 510 nm for protein-bound lutein and zeaxanthin, respectively [22,23]. Therefore we can indeed distinguish between the two carotenoids in *ex vivo* macular tissue by choosing the appropriate excitation wavelength, taking advantage of their different absorption properties.

### Conformation of macular carotenoids

The ν_4_ region of carotenoid Raman spectra arises from out-of-plane wagging vibrations of the C-H groups along the chain. As the absorption transition of carotenoids is oriented *along* the conjugated plane of the molecule, these *out-of-plane* motions will not be coupled with the transition for a perfectly-planar carotenoid. Thus the ν_4_ modes are formally resonance-forbidden for such a planar carotenoid, and isolated carotenoids *in vitro* exhibit only a broad, featureless envelope of modes in this region (see Fig 2). Distortions of the molecule out of the plane result in an increase in coupling of these motions with the transition, such that these bands gain intensity in resonance spectra. They can thus be used as a fingerprint for distortions of the carotenoid backbone. In Raman spectra of the retina, this region has greater intensity than for the isolated pigments and, in particular, an increase in structure (Fig 3, left inset). This distortion of the carotenoids *in situ* is indicative of binding of the pigments to a specific locus, most probably to specific binding proteins. This is fully consistent with experiments conducted in other groups, which have indicated that the macular pigments are bound by specific xanthophyll-binding proteins [22,23]. Note, in addition, that the structure in the ν_4_ region is not the same at each wavelength–the band positions and overall shape in this region are not the same. This observation confirms that different carotenoid populations dominate the spectrum for each wavelength, as concluded from the ν_1_ position above. It also indicates that these two populations are bound in different, non-equivalent binding sites, as the constraints felt by the carotenoid must be different in each case to account for this difference in the shape of ν_4_.

It is interesting to note that the shift in ν_1_ position discussed earlier, for the different excitation wavelengths used, is greater than that observed for the isolated carotenoids. Lutein and zeaxanthin *in vitro* exhibit a ν_1_ frequency of 1526.5 and 1525 cm^-1^ respectively (Fig 2), a shift of 1.5 cm^-1^, whereas this band shifts 3.5 cm^-1^, from 1525 to 1521.5 cm^-1^, in retinal tissue (Figs 3 and 4). This is consistent with the larger shift in absorption transition observed for the reconstituted proteins– 18 nm, from 482 to 510 nm [22,23]. It therefore appears that the zeaxanthin binding locus exerts a greater influence on its electronic properties than does the binding site for lutein. Once again this indicates that the two carotenoid binding pockets are not equivalent, and lends further weight to the conclusion that these pigments are bound in specific (and different) sites in different proteins. Access to both the absorption position and ν_1_ frequency of carotenoid molecules allows for the determination of those parameters which tune their absorption (and other electronic properties) [17,25]. Assuming that lutein and zeaxanthin absorption in the macula corresponds with those determined for the reconstituted proteins, we would conclude that the lutein binding site possesses a polarisability equivalent to THF, but extends its conjugation length by bringing the conjugated end-cycle more into the plane of the C = C chain, while that of zeaxanthin exhibits a higher polarisability value (closer to that of CS_2_). It is interesting to speculate on the role of this differential influence on the properties of the macular pigment. It is possible that these differences occur in order to tune the absorption properties of the two macular carotenoids, shifting them further apart in order to increase their complementary absorption cross-section. This would in turn increase the efficiency of blue-light filtering by the macular pigment. On the other hand, the position of the electronic energy levels of each carotenoid, reflected by the absorption and Raman properties measured here, will also have a direct influence on the putative anti-oxidant roles of MP. All of the photoprotective functions of carotenoids in photosynthesis are mediated by their electronic properties, and so if (for instance) MP has a role in preventing peroxidation reactions, as evidenced elsewhere [14], then such anti-oxidant functions should also be modulated by the changes revealed here.

### Resonance Raman as a measure of MP levels

Resonance Raman spectroscopy has already been used for the detection and quantification of carotenoids in live patients, notably in the retina [19,20]. The results presented here show that this technique can provide further information, as it is capable of distinguishing between the carotenoid species present (lutein and zeaxanthin). However, the optical properties of the eye can have an adverse affect on the measured Raman signal in live patients. In particular, lens opacity and the presence of cataracts can both result in significant scattering artefacts which reduce the measured Raman intensity, leading to an under-estimation of MP levels [26]. This is of particular concern as these optical defects are more prevalent in older patients, where accurate MP measurement is of greater importance (as the likelihood of developing macular degeneration increases with age). This has therefore proved to be a major drawback in the use of this technique for MP quantification.

As well as the macular carotenoid, the retinal pigment bound by photoreceptor opsins also has a significant Raman cross-section, and is similarly subject to the resonance phenomenon [27]. We should therefore expect signals from the retinal in photoreceptor cells to be present in Raman spectra of the intact retina. It should be noted, however, that upon removal of the retina from the eye, a significant proportion of the photoreceptor opsins are bleached by ambient light. It is therefore not clear to what extent (if any) retinal signals will still be observed in the spectra of *ex vivo* retinal tissue measured here. Note also that some of the retinal signal may become bleached during the measurement, so a quantitative relationship between individual spectra may not be observed for the *ex vivo* tissue used in this study.

In Fig 4, resonance Raman spectra in the ν_1_ region are shown for the macula at 488 and 514.5 nm excitation, at both room temperature and 77 K. Two differences are observed upon decreasing the temperature. Firstly, narrowing of the absorption bands of lutein and zeaxanthin at 77 K results in better selectivity between them. This has the effect of narrowing the ν_1_ band for each excitation wavelength, as the contributions of one carotenoid dominate the spectra to a greater extent in each case, with reduced contributions from the other pigment. Secondly the frequency of the ν_1_ mode is upshifted at the lower temperature by about 5 cm^-1^. This temperature dependence of the ν_1_ position has already been observed for carotenoids *in vitro* and when bound to protein, and was explained in terms of an intrinsic sensitivity of the mode to temperature [28]. Other than these temperature-related differences, the main features observed in this region at both temperatures are those already described above. However, there also appears a small but significant shoulder on the high frequency side of the ν_1_ band at both excitations (indicated by full arrows; more evident at low temperature due to the reduction in bandwidth). At 488 nm this shoulder appears around 1540–1545 cm^-1^ (blue full arrow)—as expected for rhodopsin, the photoactive protein in rod photoreceptors [27]. Conversely at 514.5 nm, the shoulder is observed around 1530–1535 cm^-1^ (black arrow), the ν_1_ frequency of the opsin in green cones [27]. While these retinal signals are much smaller than that from the carotenoids present, they are nevertheless observable, and it is probable that in live macular tissue (where opsins are constantly recycled) they will be significantly higher. They therefore represent a potential target for normalisation of the overall Raman signal. This should allow us to circumvent the problems described above, for the estimation of MP levels in subjects exhibiting large scattering artefacts, as the retinal signals will be reduced to the same extent by these artefacts.

Fig 4 also illustrates the effect of scanning in towards the central part of the fovea (for 488-nm excitation; blue dotted lines). The small shoulder around 1520–1525 cm^-1^ clearly increases for measurements closer to the centre (blue dotted arrow, Fig 4). By comparison with the 514.5-nm-excited spectrum, this shoulder can be attributed to minor zeaxanthin contributions at this wavelength. Thus the more central the region analysed, the greater the contribution of zeaxanthin that is observed in the Raman spectrum. This is a very clear demonstration of the validity of this method for quantifying MP and for discriminating between lutein and zeaxanthin. Indeed, this increase in zeaxanthin contribution correlates very well with the increase in zeaxanthin:lutein ratio observed by HPLC analyses when scanning in towards the central foveal region [2].

## Conclusions

The results presented here demonstrate the potential of resonance Raman spectroscopy for analyzing macular pigment structure in the human retina. Raman signals from retinal carotenoids have been measured *in situ*, and they can be used to distinguish between the different carotenoid species present (lutein and zeaxanthin). This discrimination is possible because of the difference in conjugated chain length for these two carotenoids, reflected in the position of their absorption transition and their Raman ν_1_ band.

Analysis of the ν_4_ region has allowed us to determine that both carotenoids are present in the macular in a constrained conformation, consistent with their probable binding to protein hosts [13]. The finer details of these binding phenomena are different for each carotenoid, indicating that the binding pocket is different and non-equivalent in each case. This is also reflected by a differential influence of the proteins on the functional properties of the bound pigment, with zeaxanthin exhibiting a larger shift in absorption transition and ν_1_ position.

Finally, we have re-assessed the possible use of resonance Raman spectroscopy for the quantification of MP levels in live patients. This is of particular interest as Raman has the potential to provide far more information than merely the MP density, as discussed here. We show that the presence of small but significant signals from retinal opsins should provide a means for normalization of the signal coming from the macular carotenoid.

## References

[1] Soemmering P, Home E. An Account of the Orifice in the Retina of the Human Eye, Discovered by Professor Soemmering. To Which are Added, Proofs of This Appearance Being Extended to the Eyes of Other Animals. By Everard Home, Esq. F. R. S. Philos Trans R Soc Lond. 1798;88: 332–345. 10.1098/rstl.1798.0013

[2] Landrum JT, Bone RA. Lutein, zeaxanthin, and the macular pigment. Arch Biochem Biophys. 2001;385: 28–40. 11361022 10.1006/abbi.2000.2171

[3] Sharifzadeh M, Zhao D-Y, Bernstein PS, Gellermann W. Resonance Raman imaging of macular pigment distributions in the human retina. J Opt Soc Am A. 2008;25: 947–957. 10.1364/JOSAA.25.000947 PMC3079576 18382494

[4] Snodderly DM, Auran JD, Delori FC. The macular pigment. II. Spatial distribution in primate retinas. Invest Ophthalmol Vis Sci. 1984;25: 674 6724837

[5] Kijlstra A, Tian Y, Kelly ER, Berendschot TTJM. Lutein: More than just a filter for blue light. Prog Retin Eye Res. 2012;31: 303–315. 10.1016/j.preteyeres.2012.03.002 22465791

[6] Bone RA, Landrum JT, Hime GW, Cains A, Zamor J. Stereochemistry of the human macular carotenoids. Invest Ophthalmol Vis Sci. 1993;34: 2033–2040. 8491553

[7] Li B, Ahmed F, Bernstein PS. Studies on the singlet oxygen scavenging mechanism of human macular pigment. Arch Biochem Biophys. 2010;504: 56–60. 10.1016/j.abb.2010.07.024 20678467 PMC2957523

[8] Demmig-Adams B. Carotenoids and photoprotection in plants: A role for the xanthophyll zeaxanthin. Biochim Biophys Acta BBA—Bioenerg. 1990;1020: 1–24. 10.1016/0005-2728(90)90088-L

[9] Ruban A, Berera R, Ilioaia C, van Stokkum I, Kennis J, Pascal A, et al Identification of a mechanism of photoprotective energy dissipation in higher plants. Nature. 2007;450: 575–579. 10.1038/nature06262 18033302

[10] Gall A, Berera R, Alexandre MTA, Pascal AA, Bordes L, Mendes-Pinto MM, et al Molecular Adaptation of Photoprotection: Triplet States in Light-Harvesting Proteins. Biophys J. 2011;101: 934–942. 10.1016/j.bpj.2011.05.057 21843485 PMC3175079

[11] Johnson M, Havaux M, Triantaphylides C, Ksas B, Pascal A, Robert B, et al Elevated zeaxanthin bound to oligomeric LHCII enhances the resistance of Arabidopsis to photooxidative stress by a lipid-protective, antioxidant mechanism. J Biol Chem. 2007;282 10.1074/jbc.M702831200 17553786

[12] Frank HA, Young AJ, Britton G, Cogdell RJ. The photochemistry of carotenoids [Internet]. Dordrecht, Netherlands; Boston: Kluwer Academic; 1999 Available: http://link.springer.com/book/10.1007%2F0-306-48209-6.

[13] Yemelyanov AY, Katz NB, Bernstein PS. Ligand-binding characterization of xanthophyll carotenoids to solubilized membrane proteins derived from human retina. Exp Eye Res. 2001;72: 381–392. 11273666 10.1006/exer.2000.0965

[14] Bhosale P, Bernstein PS. Synergistic effects of zeaxanthin and its binding protein in the prevention of lipid membrane oxidation. BBA-Mol Basis Dis. 2005;1740: 116–121. 10.1016/j.bbadis.2005.02.002 15949677

[15] Bernstein PS, Balashov NA, Tsong ED, Rando RR. Retinal tubulin binds macular carotenoids. Invest Ophthalmol Vis Sci. 1997;38: 167–175. 9008641

[16] Loane E, Nolan JM, O’Donovan O, Bhosale P, Bernstein PS, Beatty S. Transport and Retinal Capture of Lutein and Zeaxanthin with Reference to Age-related Macular Degeneration. Surv Ophthalmol. 2008;53: 68–81. 10.1016/j.survophthal.2007.10.008 18191658

[17] Mendes-Pinto MM, Sansiaume E, Hashimoto H, Pascal AA, Gall A, Robert B. Electronic Absorption and Ground State Structure of Carotenoid Molecules. J Phys Chem B. 2013;117: 11015–11021. 10.1021/jp309908r 23294447

[18] Robert B, Horton P, Pascal A, Ruban A. Insights into the molecular dynamics of plant light-harvesting proteins in vivo. Trends Plant Sci. 2004;9: 385–390. 10.1016/j.plants.2004.06.006 15358269

[19] Ermakov I, Bernstein PS, Ermakova M, Gellermann W. Macular pigment Raman detector for clinical applications. J Biomed Opt. 2004;9: 139–148. 10.1117/1.1627776 14715066 PMC3086335

[20] Gellermann W, Ermakov IV, Ermakova MR, McClane RW, Zhao D-Y, Bernstein PS. In vivo resonant Raman measurement of macular carotenoid pigments in the young and the aging human retina. J Opt Soc Am A. 2002;19: 1172–1186. 10.1364/JOSAA.19.001172 12049355

[21] Ruban AV, Pascal AA, Robert B, Horton P. Configuration and Dynamics of Xanthophylls in Light-Harvesting Antennae of Higher Plants. Spectroscopic Analysis of Isolated Light-Harvesting Complex of Photosystem II and Thylakoid Membranes. J Biol Chem. 2001;276: 24862–24870. 10.1074/jbc.M103263200 11331293

[22] Bhosale P, Larson AJ, Frederick JM, Southwick K, Thulin CD, Bernstein PS. Identification and Characterization of a Pi Isoform of Glutathione S-Transferase (GSTP1) as a Zeaxanthin-binding Protein in the Macula of the Human Eye. J Biol Chem. 2004;279: 49447–49454. 10.1074/jbc.M405334200 15355982

[23] Li B, Vachali P, Frederick JM, Bernstein PS. Identification of StARD3 as a Lutein-Binding Protein in the Macula of the Primate Retina. Biochemistry (Mosc). 2011;50: 2541–2549. 10.1021/bi101906y PMC3070171 21322544

[24] Phillip D, Ruban AV, Horton P, Asato A, Young AJ. Quenching of chlorophyll fluorescence in the major light-harvesting complex of photosystem II: a systematic study of the effect of carotenoid structure. Proc Natl Acad Sci. 1996;93: 1492–1497. 11607629 10.1073/pnas.93.4.1492 PMC39967

[25] Macernis M, Sulskus J, Malickaja S, Robert B, Valkunas L. Resonance Raman Spectra and Electronic Transitions in Carotenoids: A Density Functional Theory Study. J Phys Chem A. 2014; 10.1021/jp406449c 24527866

[26] Howells O, Eperjesi F, Bartlett H. Measuring macular pigment optical density in vivo: a review of techniques. Graefes Arch Clin Exp Ophthalmol. 2011;249: 315–347. 10.1007/s00417-010-1577-5 21221629

[27] Kochendoerfer GG, Lin SW, Sakmar TP, Mathies RA. How color visual pigments are tuned. Trends Biochem Sci. 1999;24: 300–305. 10.1016/S0968-0004(99)01432-2 10431173

[28] Andreeva A, Apostolova I, Velitchkova M. Temperature dependence of resonance Raman spectra of carotenoids. Spectrochim Acta A Mol Biomol Spectrosc. 2011;78: 1261–1265. 10.1016/j.saa.2010.12.071 21269874

